# Delivering bad news fairly: The influence of core self-evaluations and anxiety for the enactment of interpersonal justice

**DOI:** 10.1177/00187267211011000

**Published:** 2021-04-17

**Authors:** Annika Hillebrandt, Maria Francisca Saldanha, Daniel L Brady, Laurie J Barclay

**Affiliations:** Ryerson University, Canada, ahillebrandt@ryerson.ca; Universidade Católica Portuguesa, Portugal, mfsaldanha@ucp.pt; Wilfrid Laurier University, Canada, dbrady@wlu.ca; Wilfrid Laurier University, Canada, lbarclay@wlu.ca

**Keywords:** appraisal theory, core self-evaluations, emotions, interpersonal justice, justice enactment

## Abstract

What motivates managers to deliver bad news in a just manner and why do some managers fail to treat recipients of bad news with dignity and respect? Given the importance of delivering bad news in a just manner, answering these questions is critical to promote justice in the workplace. Drawing on appraisal theories of emotions, we propose that people with higher core self-evaluations may be less likely to deliver bad news in an interpersonally just manner. This is because these actors are more likely to appraise the delivery of bad news as a situation in which they have high coping potential and are therefore less likely to experience anxiety. However, we propose that anxiety can be important for propelling the enactment of interpersonal justice. We test our predictions across three studies (with four samples of full-time managers and employees). Theoretical and practical contributions include enhancing our understanding of *who* is motivated to enact interpersonal justice, *why* they are motivated to do so, and *how* to enhance justice in the workplace. Our findings also challenge the assumption that negative emotions are necessarily dysfunctional for the enactment of interpersonal justice and instead highlight the facilitative role of anxiety in this context.

Good people do bad things when they fail to see as they should. (Folger and Skarlicki, 2001: 102)

The best antidote to anxiety isn’t calm. It isn’t distraction. It’s action. (Grant, 2020)

Delivering bad news is a critical part of life in organizations. While actors (e.g. managers) often loathe performing this task and worry about its consequences, extensive research has shown that those who deliver bad news in a manner that displays dignity and respect for the recipient (i.e. enact interpersonal justice) can mitigate damage and promote acceptance of the news (e.g. Bies, 2013; Folger and Skarlicki, 1998, 2001). Despite these benefits, people often fail to enact interpersonal justice when delivering bad news. While emerging evidence suggests that characteristics of the actor can be related to interpersonal justice (e.g. Patient and Skarlicki, 2010; Whiteside and Barclay, 2016), *why* these effects emerge remains unclear. Addressing these issues is critical for providing insights into how these negative effects can be overcome. That is, identifying *who* is less likely to enact justice and *why* these effects occur can provide a deeper theoretical understanding that can be used to more effectively promote justice in the workplace.

Drawing upon appraisal theories (e.g. Lazarus, 1991), we argue that core self-evaluations (i.e. a stable personality trait that reflects fundamental evaluations of oneself; Judge et al., 1998) can impact the enactment of interpersonal justice. Our general argument is that actors with higher core self-evaluations are less likely to enact interpersonal justice because they are more confident in their own coping potential (i.e. perceived ability to manage or ameliorate the situation; Lazarus, 1991) and are therefore less likely to experience anxiety about delivering bad news. However, we propose that anxiety is important in this situation because anxiety can propel actors to engage in behaviors to reduce or manage the potential threat to one’s social esteem that can be inherent in situations involving the delivery of bad news. That is, anxiety can prompt the enactment of interpersonal justice (i.e. adherence to justice rules related to providing dignity and respect). Taken together, although core self-evaluations are often positively related to managerial effectiveness (e.g. Judge and Kammeyer-Mueller, 2011), we propose that this trait is *negatively* associated with the enactment of interpersonal justice. Figure 1 displays our model.

**Figure 1.**
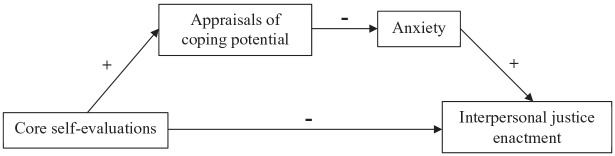
Theoretical model.

We aim to make three primary theoretical contributions. First, scholars have argued that it is critical to motivate actors to promote interpersonal justice (e.g. Ambrose and Schminke, 2009; Scott et al., 2009), especially given its pervasive benefits for employees and organizations (see Colquitt et al., 2013 for a meta-analytic review). However, there is some evidence that not all actors are motivated to enact interpersonal justice (e.g. managers with low trait empathy; Patient and Skarlicki, 2010; Whiteside and Barclay, 2016). Drawing on appraisal theories, we identify core self-evaluations as being important for the enactment of interpersonal justice, such that this personality characteristic may negatively influence actors’ motivation to engage in this behavior. By examining core self-evaluations, we highlight how a characteristic that is typically associated with managerial effectiveness (e.g. Erez and Judge, 2001) may have the counterintuitive effect of undermining actors’ motivation to enact interpersonal justice.

Second, while identifying *who* is motivated to enact interpersonal justice is important, it is also critical to understand *why* they are motivated to do so. Drawing on appraisal theories of emotion (e.g. Lazarus, 1991), we argue that core self-evaluations are negatively associated with the enactment of interpersonal justice because this managerial characteristic can impact actors’ appraisals of their coping potential related to the delivery of bad news. In turn, these appraisals can predict the extent to which actors experience anxiety, which can motivate the enactment of interpersonal justice. Thus, we identify appraisals of coping potential and anxiety as sequential mechanisms underlying the relationship between managerial characteristics (i.e. core self-evaluations) and the enactment of interpersonal justice. In doing so, we highlight *why* people can be motivated to enact interpersonal justice as well as identify points of intervention to enhance the enactment of interpersonal justice.

Third, current recommendations within the justice enactment literature focus on the importance of curtailing managers’ negative affect to promote justice behaviors (e.g. Scott et al., 2014). This recommendation is based on the finding that ‘negative affect’ (operationalized as anger) is associated with decreased enactment of interpersonal justice. However, appraisal theories indicate that it is critical to recognize distinctions between negative emotions (e.g. anger versus anxiety) because these emotions serve disparate functions for the individual and can also be differentially related to behaviors (Lazarus, 1991). Given the nature of anxiety, we argue that this emotion can have facilitative effects in the context of the delivery of bad news, such that anxiety can *increase* managers’ motivation to enact interpersonal justice. By recognizing the facilitative role of anxiety for the enactment of interpersonal justice, we highlight the importance of recognizing distinctions between negative emotions (e.g. anxiety versus anger) and challenge the assumption that negative emotions are inherently dysfunctional for interpersonal justice.

## Theoretical background

Appraisal theories of emotion indicate that people appraise situations to determine the potential implications for their well-being (Lazarus, 1991). Appraisals have two components: primary and secondary appraisals. Primary appraisals reflect the relevance of the situation for one’s own well-being and consider the person within the context of their roles and relationships (i.e. their ego involvement; Lazarus, 1991). For example, in the context of bad news delivery, actors may be especially concerned about their social esteem (i.e. maintaining a fair identity, such that they are viewed by themselves and/or others as a fair person; Lerner, 1980; Scott et al., 2014; Tripp and Bies, 2009) because this can have implications for their well-being.

Situations that are appraised as being likely to hinder or advance one’s well-being can elicit negative versus positive emotions, respectively. However, secondary appraisals are needed to generate specific discrete emotions. More precisely, discrete emotions may reflect the nature of one’s ego involvement in the situation and one’s perceived ability to cope with the situation (e.g. ‘what, if anything, can I do in this encounter’; Lazarus, 1991: 134). For example, anxiety is likely to arise when the situation highlights the potential for harm to one’s social esteem and one’s coping potential is perceived to be uncertain. By contrast, anger typically arises when the situation highlights that someone else is to blame for one’s goals being hindered. In turn, discrete emotions can prompt behaviors that enable people to adapt to their environment (e.g. anxiety can motivate people to address the potential for harm and/or protect their own interests).

Appraisal theories indicate that people not only appraise current situations, but also appraise anticipated or imagined situations (Folkman and Lazarus, 1985). For example, actors who know that they need to deliver bad news to another person may anticipate and appraise this situation. Importantly, people’s personality characteristics have been theorized and empirically shown to shape these appraisals. That is, personality characteristics can serve as ‘causal antecedents’ that can impact appraisals and therefore individuals’ emotional and behavioral reactions (Lazarus, 1991: 209–210; also see Lazarus and Folkman, 1984; Smith and Lazarus, 1990; Smith and Pope, 1992).

Applying these theoretical tenets, our general argument is that core self-evaluations are likely to be negatively related to the enactment of interpersonal justice because core self-evaluations can influence the appraisal process. That is, core self-evaluations can impact appraisals of coping potential and therefore anxiety – an important driver of interpersonal justice enactment. Below, we build our theoretical argument that core self-evaluations are negatively related to the enactment of interpersonal justice via appraisal processes. We begin by outlining how core self-evaluations can influence appraisals of coping potential.

### Core self-evaluations and appraisals of coping potential

We propose that core self-evaluations can influence how actors appraise a social situation in which they need to deliver bad news to another person. Core self-evaluations capture the ‘fundamental premises that individuals hold about themselves and their functioning in the world’ (Judge et al., 1998: 168), with higher core self-evaluations reflecting higher self-esteem (i.e. the value one places on oneself), higher self-efficacy (i.e. beliefs in one’s ability to succeed), an internal locus of control (i.e. the belief that events are caused by internal forces), and lower neuroticism (i.e. a reduced tendency to exhibit poor emotional adjustment) (Judge et al., 2002). Thus, people with higher core self-evaluations are more likely to believe that they are in control of their environment, more confident that they are capable of solving their own problems, and less likely to focus on negative aspects of a situation or experience doubt (e.g. Judge and Kammeyer-Mueller, 2011).

We argue that core self-evaluations can impact people’s appraisals of their coping potential related to the anticipated delivery of bad news. In general, delivering bad news can reflect a situation that is potentially threatening to one’s social esteem because actors often expect that the recipient will evaluate them negatively or reject them (Rosen and Tesser, 1972; Tesser and Rosen, 1975), even if the actor was not personally responsible for creating the circumstances that led to the negative outcome (e.g. Lavelle et al., 2016). That is, actors can be concerned that the recipient will ‘shoot the messenger’ (Tripp and Bies, 2009). This can be perceived as threatening to actors because people are typically motivated to maintain a fair identity (e.g. Barclay et al., 2017; Lerner, 1980), especially because this social identity can convey psychological benefits (e.g. esteem; Scott et al., 2014) as well as a range of positive outcomes (e.g. enhanced legitimacy, social relationships; Greenberg, 1990; Long, 2016).

However, appraisal theories indicate that people not only consider the implications of the situation for their well-being, but also consider their ability to manage or ameliorate the situation (i.e. their coping potential) when appraising the situation (Lazarus, 1991). Core self-evaluations have been theorized to enhance appraisals of coping potential in social situations – that is, those with higher core self-evaluations may perceive that they have greater control over other people’s actions and perceptions, owing to their ‘enhance[d] . . . sense of control over things, persons, and elements in the environment’ (Oh and Farh, 2017: 215). That is, actors with high core self-evaluations may perceive that they can effectively manage the situation. Thus, we propose that people with higher core self-evaluations should perceive that they will be better able to cope with this situation (i.e. have higher appraisals of coping potential), given their enhanced sense of personal agency and control, positive sense of self-worth, and greater emotional stability.

### Appraisals of coping potential and the elicitation of anxiety

As noted above, anxiety can be elicited by situations in which the person is concerned about the ‘protection of personal meaning or ego-identity’ and is also uncertain about what may happen (Lazarus, 1991: 237).^1^ That is, recognizing that one must deliver bad news may prompt the actor to appraise the potential for loss or harm to their social esteem in this situation. People tend to experience anxiety when they are unsure whether they will be able to cope with a situation that is potentially threatening to their social esteem. This suggests that anxiety may be experienced by those who must deliver bad news to others because this situation may raise concerns about maintaining one’s identity as a fair person. However, appraisal theories also indicate that people incorporate information related to their coping potential and the outcome of this secondary appraisal process is critical for the elicitation of discrete emotions (i.e. anxiety). More precisely, people who perceive that they can effectively manage or ameliorate the situation are less likely to experience anxiety (Lazarus, 1991). In other words, people who perceive that they have high coping potential about delivering bad news to another person should be less likely to experience anxiety about this situation. This is because high coping potential can make the situation seem less threatening and/or less likely to require the protection of one’s social esteem.

### Anxiety and the enactment of interpersonal justice

Appraisal theories of emotion indicate that specific discrete emotions (e.g. anxiety) can motivate behaviors that are intended to help the individual navigate or adapt to the situation (Lazarus, 1991). Given that anxiety focuses on the presence of a potential threat to one’s social esteem, anxiety can motivate individuals to reduce, eliminate, or protect themselves from this potential threat (Spielberger, 1985). However, the uncertainty associated with anxiety may also make it difficult to determine an appropriate course of action. Not surprisingly, anxiety has been shown to have contrasting effects in that it can motivate withdrawal or approach behaviors as well as have debilitative or facilitative effects (for a detailed discussion of moderators for these effects, see Cheng and McCarthy, 2018). Importantly, studies have shown that anxiety can have *facilitative* effects if one’s behavior can reduce the potential threat. For example, Barclay and Kiefer (2019) found that anxiety can motivate employees to engage in problem-prevention behaviors to fix problems related to an unfair event.

Within the context of delivering bad news, this suggests that people may try to avoid the threat, where possible. For example, a manager may avoid making the decision about whether layoffs should occur (i.e. avoid the preparation stage of bad news delivery). However, once the decision has been made and the manager is implementing the decision (i.e. in the delivery stage), we argue that anxiety can have facilitative effects. More precisely, once managers enter the delivery stage, they are unable to avoid the threat so the best course of action in this situation is to try to reduce the threat. As such, anxiety should enhance people’s arousal and energize them to engage in behaviors that can reduce potential harm (Carver and Scheier, 1988; Cheng and McCarthy, 2018). Thus, we argue that anxiety can motivate actors to enact interpersonal justice when delivering bad news because these behaviors can reduce potential harm by helping recipients feel more fairly treated and promoting their acceptance of the bad news (e.g. Bies, 2013; Folger and Skarlicki, 1998, 2001) as well as by maintaining or validating the actor’s identity as a fair person (e.g. Scott et al., 2009, 2014). Taken together, while anxiety may not always have facilitative effects, we argue that the delivery of bad news is a situation in which anxiety is likely to have a facilitative role because there is a clear behavior that can reduce threat (i.e. the enactment of interpersonal justice can reduce the potential harm to the actor’s esteem).

Integrating the above, we propose that core self-evaluations are negatively related to the enactment of interpersonal justice and that this relationship can be explained by appraisals of coping potential and anxiety. That is, actors’ core self-evaluations should influence their appraisals of coping potential, such that actors with higher core self-evaluations should perceive that they have higher coping potential. In turn, appraisals of coping potential should be negatively associated with anxiety. However, anxiety is important because it can motivate the enactment of interpersonal justice. Taken together, given that people with higher core self-evaluations have higher appraisals of coping potential and are therefore less likely to experience anxiety, we propose that they should be less inclined to enact interpersonal justice. Thus, we predict that core self-evaluations negatively predict interpersonal justice enactment and that this effect is sequentially mediated by appraisals of coping potential and anxiety (see Figure 1).

Given that our argumentation focuses on how core self-evaluations can impact the enactment of interpersonal justice via appraisal processes, we formally hypothesize three key mediations to focus on the mechanisms underlying these relationships. More precisely, given that a core tenet of appraisal theories of emotions is that emotions are a key mechanism driving behavior, we first focus on the mediating role of anxiety in the relationship between core self-evaluations and the enactment of interpersonal justice (H1). Next, we delve deeper into the appraisal process by predicting that appraisals of coping potential can serve as a key mechanism linking core self-evaluations to anxiety (H2). Finally, we examine the full appraisal process by predicting that the relationship between core self-evaluations and interpersonal justice is serially mediated by appraisals of coping potential and anxiety (H3).

*Hypothesis 1*: Core self-evaluations have a negative indirect effect on interpersonal justice enactment via anxiety.
*Hypothesis 2*: Appraisals of coping potential mediate the negative effect of core self-evaluations on anxiety.
*Hypothesis 3*: The relationship between core self-evaluations and interpersonal justice enactment is serially mediated by appraisals of coping potential and anxiety.

### Overview of studies

We examine the delivery of bad news (e.g. layoffs and promotion denials) because the enactment of interpersonal justice can be especially important in this context. More precisely, the delivery of bad news is a multi-phase process involving preparation (e.g. deciding who will be laid off), delivery of the news (e.g. communicating the layoff to the recipient), and transition (e.g. helping recipients move forward; for a layoff, this may involve such activities as providing reference letters or connections to an outplacement assistance program whereas promotion denials may involve providing training opportunities). We focus on the *delivery* phase because this is where interpersonal justice is likely to be most critical for recipients (see Bies, 2013).

We test our theoretical arguments across three studies. We begin our investigation by focusing on the mediating role of anxiety in the relationship between core self-evaluations and the enactment of interpersonal justice (H1) in Studies 1a and 1b. Next, we explicitly test appraisals of coping potential as the mechanism relating core self-evaluations to anxiety (H2). More precisely, Study 2 uses a moderation-of-process design (see Spencer et al., 2005; Vancouver and Carlson, 2015) in which appraisals of coping potential are manipulated to test their mediating role in the relationship between core self-evaluations and anxiety (H2) as well as their temporal ordering in the serial mediation (H3). Using a measured variable approach, Study 3 re-tests our serial mediation (H3). Together, these studies examine the mediating role of appraisals of coping potential and anxiety as well as the temporal ordering of these mechanisms in the relationship between core self-evaluations and the enactment of interpersonal justice.

## Study 1

Study 1 tests the indirect relationship between core self-evaluations and interpersonal justice enactment via anxiety. To enhance generalizability, we examine our relationships in two disparate bad news contexts with different samples. Whereas Study 1a focuses on a sample of full-time managers and uses a layoff context, Study 1b focuses on a heterogeneous sample of managers/full-time employees and focuses on a promotion denial. Both contexts are commonly researched ‘necessary evils’ in the management literature (Molinsky and Margolis, 2005). To provide confidence that the effects are being driven by core self-evaluations rather than other theoretically relevant characteristics or experiences of the actor, we also examine sense of power, the dark triad, and prior layoff experiences (i.e. having laid off others and having been laid off oneself; Study 1a) as well as trait empathy and supervisor position (Study 1b).

Prior to conducting Studies 1a and 1b, we conducted a pilot study (*N* = 98) that tested the effect of core self-evaluations on interpersonal justice enactment. The procedures for the pilot study were identical to Study 1a (below). The effect size for the relationship between core self-evaluations and interpersonal justice enactment in this pilot study was *R*^2^ = .05. Based on this estimated effect size of *R*^2^ = .05, a 95% confidence interval, a desired power of .80, and two predictors (i.e. core self-evaluations and anxiety), we calculated a priori sample sizes of 187 for Studies 1a and 1b. However, we recruited slightly larger sample sizes to account for potential missing data and incomplete surveys.^2^

### Study 1a participants and procedure

Full-time managers from North America (*N* = 225) were recruited using Prolific (see Palan and Schitter, 2018; Peer et al., 2017). A total of 224 participants completed the study. Participants were paid US$3.25. We followed best practices to ensure the quality of the online data, including screening for inattentiveness using two attention checks (e.g. ‘select agree to respond to this question’; see Cheung et al., 2017).^3^ Five participants were removed from the analyses for failing to follow the communication task instructions (e.g. failing to provide a response on the task or failing to provide an understandable response that could be coded). The final sample (*N* = 219) was 42% female with a mean age of 34.97 years (*SD =* 8.96), work experience of 15.04 years (*SD* = 9.25), tenure in their organization of 6.64 years (*SD* = 5.07), and 9.48 subordinates (*SD* = 14.48).

Participants completed a communication task validated by Patient and Skarlicki (2010) in which they had to communicate a layoff decision to an employee (‘Bob’). The scenario was designed such that the organization, the manager, and the employee each held some degree of accountability for the layoff so that a range of communication strategies would be reasonable.

Immediately after reading the scenario, participants were asked to imagine that they would soon be meeting with Bob in person to communicate the layoff decision. Next, they completed a measure assessing anxiety in anticipation of this meeting. Finally, participants were given the following instructions: Imagine that Bob has agreed to meet you in your office. You now need to communicate the layoff decision to Bob. Below, please write down what you want to say. Imagine you are actually communicating the layoff decision to Bob.Following this task, participants completed filler items followed by our trait measure of core self-evaluations.

### Study 1a measures

*Anxiety* was assessed with two items from Folkman and Lazarus (1985; ‘anxious’, ‘worried’). Participants were instructed to ‘respond to each statement below based on your feelings about this upcoming meeting with Bob’. The response scale ranged from *not at all* (1) to *very much* (7). *Interpersonal justice enactment* was assessed following procedures from Patient and Skarlicki (2010). Two independent coders who were blind to the responses on the rest of the survey coded the written messages for interpersonal justice by assessing the extent to which the communicator was polite and courteous, treated the employee with dignity and respect, and expressed concern for the employee. The rating scale ranged from *not at all* (1) to *very much* (5). The intraclass correlation coefficient (ICC_2_) was .89, indicating excellent intercoder agreement (see Koo and Li, 2016). Discrepancies were identified and then resolved through discussion. The mean word count of the written messages was 118.30 (*SD* = 66.57; min. = 19; max. = 449). *Core self-evaluations* were assessed with Judge et al.’s (2003) scale (12 items; e.g. ‘When I try, I generally succeed’). The response scale ranged from *strongly disagree* (1) to *strongly agree* (5).

#### Control variables

We assessed several other variables to rule out alternative theoretical explanations for the hypothesized relationships. These variables were not included in our main analyses but rather in supplemental analyses. We assessed *sense of power* with Anderson and Galinsky’s (2006) scale (eight items; e.g. ‘I can get people to listen to what I say’) because those with higher power have been theorized to be less likely to enact interpersonal justice (i.e. failing to enact justice is less risky for them; Scott et al., 2014). Second, we measured the *dark triad* traits (i.e. Machiavellianism, narcissism, and psychopathy) with Jonason and Webster’s (2010) scales (four items per trait; e.g. ‘I tend to manipulate others to get my way’) because people who are high on dark triad personality traits have a tendency to be callous, selfish, and malevolent when interacting with others (Paulhus and Williams, 2002). Response scales for sense of power and the dark triad ranged from *strongly disagree* (1) to *strongly agree* (5). Finally, we assessed prior experiences with layoffs because this may impact one’s motivation to enact justice. Participants were asked if they had ever *conducted a layoff* (*yes* = 1, *no* = 2) or *been laid off* (*yes* = 1, *no* = 2).

### Study 1a results and discussion

Table 1 presents the means, standard deviations, reliabilities, and correlations. A confirmatory factor analysis (CFA) on our measures of core self-evaluations, anxiety, and interpersonal justice enactment indicated adequate fit, χ²(84) = 159.31, *p* < .001; CFI = .95; RMSEA = .06.^4^ This model also had significantly better fit (*p* < .001) than an alternative measurement model in which core self-evaluations and anxiety (i.e. the two self-report variables) were combined (χ²(86) = 327.61, *p* < .001), which provides empirical evidence for the distinctiveness of our variables.

**Table 1.** Means, standard deviations, correlations, and reliabilities (Study 1a).

Variable	*M*	*SD*	1	2	3	4	5	6	7	8	9
1. Core self-evaluations	3.57	.81	(.91)								
2. Anxiety	4.95	1.39	−.26**	(.80)							
3. Interpersonal justice	2.93	1.12	−.15*	.21**	(–)						
4. Sense of power	3.58	.71	.61**	−.23**	−.07	(.88)					
5. Machiavellianism	2.18	.92	−.23**	.02	−.05	−.05	(.81)				
6. Narcissism	2.45	.87	−.11	.17*	−.10	.00	.47**	(.77)			
7. Psychopathy	2.16	.82	−.33**	.01	−.10	−.20**	.58**	.29**	(.71)		
8. Conducted a layoff	1.63	.48	−.16	.03	.09	−.10	−.02	−.08	.06	(–)	
9. Been laid off	1.66	.47	.04	−.05	−.05	.23**	−.06	−.05	−.04	.23**	(–)

*N* = 219. *p < .05; **p < .01. Reliabilities are shown on the diagonal in parentheses.

To ensure that our main analyses were not reliant on the presence of any covariates and to avoid inadvertently biasing the analyses by the presence of impotent control variables (for a discussion, see Becker et al., 2016), we conducted our main analyses *without* control variables. Table 2 displays the results of the analyses without the control variables and the supplemental analyses with the control variables.

**Table 2.** Results of main and supplemental regression analyses for Study 1a.

	Anxiety		Interpersonal justice enactment	
	Model 1	Model 2	Model 1	Model 2
*Main analysis*				
Intercept	6.54** (.41)		2.71** (.50)	
Core self-evaluations	−.45** (.11)		−.14 (.10)	
Anxiety			.15** (.06)	
*R* ^2^	.07		.05	
*Supplemental analysis*				
Intercept	6.31** (.74)	7.01** (.79)	3.84** (.62)	3.33** (.76)
Sense of power	−.48** (.13)	−.25 (.16)	−.13 (.11)	.10 (.13)
Machiavellianism	−.08 (.13)	−.12 (.13)	.08 (.11)	.07 (.11)
Narcissism	.35** (.12)	.34** (.12)	−.11 (.10)	−.17 (.10)
Psychopathy	−.12 (.14)	−.17 (.14)	−.20 (.12)	−.21 (.11)
Conducted a layoff	.11 (.20)	.05 (.19)	.24 (.16)	.18 (.16)
Been laid off	−.14 (.20)	−.11 (.20)	−.19 (.16)	−.15 (.16)
Core self-evaluations		−.36* (.15)		−.24 (.12)
Anxiety				.16** (.06)
*R* ^2^	.10	.12	.04	.10

* *p* < .05; **p < .01. Values are unstandardized path coefficients with standard error estimates in parentheses. The supplemental analysis was performed using control variables.

We conducted linear regression analyses to test the bivariate relationships. Consistent with our theorizing, core self-evaluations were negatively related to anxiety, *b* = −.45, *SE* = .11, *t*(218) = −3.95, *p* < .001, *R*^2^ = .07. Anxiety was positively associated with interpersonal justice enactment, *b* = .17, *SE* = .05, *t*(218) = 3.12, *p* = .002, *R*^2^ = .04. Core self-evaluations were negatively associated with interpersonal justice enactment, *b* = −.21, *SE* = .09, *t*(218) = −2.21, *p* = .028, *R*^2^ = .02. To test our mediation hypothesis, we used bootstrapping (10,000 resamples) with Process 3.5 Model 4 (Hayes, 2018) to calculate confidence intervals for the indirect effect of core self-evaluations on interpersonal justice enactment via anxiety (see Preacher and Hayes, 2008; Shrout and Bolger, 2002).^5^ The indirect effect was significant; indirect effect = −.07, *SE* = .03, 95% CI [−.14, −.01]. The mediation model accounted for 5.26% of the variance in interpersonal justice enactment. H1 was supported.

We conducted supplemental analyses to examine sense of power, dark triad traits, and prior layoff experiences (having conducted a layoff or been laid off) as alternative explanations. Our results remained substantively similar when controlling for these variables (see Table 2).

## Study 1b

Study 1b re-tests H1 using a promotion denial with a sample of managers and employees.

### Study 1b participants and procedure

Full-time employees (*N =* 200) from North America were recruited via Prolific and paid US$3.25. Respondents from Study 1a were prevented from participating. Fifteen participants were removed from the analyses for failing to follow the communication task instructions. The final sample (*N* = 185) was 38.9% female with an average age of 37.48 years (*SD =* 11.15), work experience of 17.15 years (*SD* = 11.97), and tenure in their organization of 7.08 years (*SD* = 6.54). Managers comprised 35.1% of the sample.

Participants were asked to read a scenario (see Appendix) and imagine themselves in the role of a manager who had to communicate to a subordinate (‘Tom’) that he would not receive a promotion. As in Study 1a, the scenario was designed so that all parties held some degree of accountability and a range of communication strategies would be reasonable. After reading the scenario, participants completed a measure of anxiety. Next, they were provided with the following instructions:

Tom has agreed to meet you in your office. You now need to communicate the promotion decision to Tom. In the box below, please write down what you want to say. Imagine you are actually communicating the decision to Tom.

Finally, participants completed filler measures followed by our measure of core self-evaluations.

### Study 1b measures

*Anxiety* and *core self-evaluations* were assessed using the same scales as Study 1a. *Interpersonal justice enactment* was rated by two independent coders following the same protocol as Study 1a. The ICC_2_ (.89) indicated a high level of agreement. Discrepancies were identified and then resolved through discussion. The mean word count of the messages written by participants was 101.38 (*SD* = 59.37; min. = 13; max. = 362). Given that our communication task involved delivering bad news to a subordinate, we asked participants to indicate whether they held a *supervisor position* within their organization (*yes* = 1, *no* = 2). We also examined trait empathy because it has been positively associated with the enactment of interpersonal justice (Patient and Skarlicki, 2010). *Trait empathy* was assessed with Wakabayashi et al.’s (2006) short form of the Empathy Quotient (22 items; e.g. ‘I really enjoy caring for other people’). The response scale ranged from *strongly disagree* (1) to *strongly agree* (5).

### Study 1b results and discussion

Table 3 presents the means, standard deviations, reliabilities, and correlations. A CFA conducted on measures of core self-evaluations, anxiety, and interpersonal justice enactment indicated good fit, χ²(84) = 175.74, *p* < .001; CFI = .93; RMSEA = .08. Our proposed measurement model also had significantly better fit (*p* < .001) than an alternative measurement model in which core self-evaluations and anxiety (i.e. the two self-report variables) were combined, χ²(86) = 313.66, *p* < .001.

**Table 3.** Means, standard deviations, correlations, and reliabilities (Study 1b).

Variable	*M*	*SD*	1	2	3	4	5
1. Core self-evaluations	3.54	.78	(.91)				
2. Anxiety	4.63	1.68	−.38**	(.87)			
3. Interpersonal justice	2.77	1.84	−.16*	.26**	(–)		
4. Supervisor position	1.62	.49	−.17**	.18**	−.02	(–)	
5. Trait empathy	3.65	.61	.47**	−.12	.03	−.15*	(.91)

*N* = 185. *p < .05; **p < .01. Reliabilities are shown on the diagonal in parentheses.

We used the same analytic strategy as in Study 1a. Table 4 presents the results of the main and supplemental analyses. Consistent with our theorizing, core self-evaluations were negatively associated with anxiety, *b* = −.83, *SE* = .15, *t*(184) = −5.58, *p* < .001, *R*^2^ = .15. Anxiety was positively associated with interpersonal justice enactment, *b* = .13, *SE* = .04, *t*(184) = 3.62, *p* < .001, *R*^2^ = .07. Core self-evaluations were negatively associated with interpersonal justice enactment, *b* = −.17, *SE* = .08, *t*(184) = −2.12, *p* = .036, *R*^2^ = .02. The indirect effect of core self-evaluations on interpersonal justice enactment via anxiety was significant; indirect effect = *–*.10, *SE* = .04, 95% CI [−.18, −.03]. The mediation model accounted for 7.04% of the variance in interpersonal justice enactment. H1 was supported. Supplemental analyses indicated that controlling for supervisor position and trait empathy did not substantively affect any results.

**Table 4.** Results of main and supplemental regression analyses for Study 1b.

	Anxiety		Interpersonal justice enactment	
	Model 1	Model 2	Model 1	Model 2
*Main analysis*				
Intercept	7.55* (.54)		2.49* (.40)	
Core self-evaluations	−.83* (.15)		−.07 (.08)	
Anxiety			.12* (.04)	
*R* ^2^	.15		.07	
*Supplemental analysis*				
Intercept	4.68* (.90)	6.17* (.89)	2.68* (.46)	2.37* (.52)
Supervisor position	.56* (.25)	.42 (.24)	−.02 (.13)	−.11 (.13)
Trait empathy	−.26 (.20)	.23 (.21)	.03 (.10)	.14 (.11)
Core self-evaluations		−.87* (.17)		−.13 (.09)
Anxiety				.12* (.04)
*R* ^2^	.04	.16	.00	.08

*p < .01. Values are unstandardized path coefficients with standard error estimates in parentheses. The supplemental analysis was performed using control variables.

### Study 1 discussion

Taken together, Studies 1a and 1b indicate that core self-evaluations, a trait that is typically associated with managerial effectiveness (e.g. Erez and Judge, 2001), are negatively associated with interpersonal justice enactment. Consistent with our theoretical argument that core self-evaluations influence interpersonal justice enactment via appraisal processes, our findings indicated that actors with higher core self-evaluations are less likely to experience anxiety in anticipation of delivering bad news to another person. Importantly, anxiety was *positively* related to the enactment of interpersonal justice, indicating that anxiety can motivate the enactment of interpersonal justice. Finally, we ruled out sense of power, the dark triad, prior experiences with layoffs, trait empathy, and supervisor position as alternative explanations.

## Study 2

In Study 2, we test our full serial mediation model (i.e. appraisals of coping potential and anxiety as sequential mediators) with a sample of full-time managers from the United Kingdom (versus North America) to enhance confidence in our theorizing and generalizability. More precisely, we use an experimental moderation-of-process design. This design experimentally manipulates a psychological process to provide evidence of its role as a mediator (see Spencer et al., 2005; Vancouver and Carlson, 2015). For our study, we experimentally manipulate our first mediator (appraisals of coping potential) and then measure our second mediator (anxiety) before participants enact interpersonal justice behaviors (the outcome). If appraisals of coping potential and anxiety are serial mediators, then manipulating appraisals of coping potential should affect the anxiety mediator, thereby ‘interrupting’ (i.e. interfering with) the mediation related to core self-evaluations, anxiety, and interpersonal justice (for an example, see Vancouver and Carlson, 2015). This would provide experimental evidence in support of the serial mediation and the temporal ordering of our mediators (i.e. appraisals of coping potential precede anxiety).

According to Lazarus (1991: 238), appraisals of coping potential reflect people’s assessment of their perceived ability to manage or ameliorate a threatening situation and can be enhanced by ‘cognitive coping efforts to think positively’. In Study 2, we manipulate appraisals of coping potential by affirming (versus not affirming, as a control condition) the actor’s coping potential. When coping potential is affirmed (i.e. in the experimental condition), managers should experience relatively low levels of anxiety regardless of their core self-evaluations. This should then reduce the ability of anxiety to mediate the relationship between core self-evaluation and the enactment of interpersonal justice. By contrast, when coping potential is not affirmed (i.e. in the control condition), anxiety should mediate the relationship between core self-evaluations and the enactment of interpersonal justice, as observed in Study 1. With this in mind, we examine a moderated mediation in which the appraisals of coping potential manipulation moderate the indirect effect of core self-evaluations on interpersonal justice enactment via anxiety by impacting the first leg of the mediation. If supported, this would provide evidence of serial mediation through an experimental manipulation (H3).

### Participants and procedure

Managers from the United Kingdom (*N =* 350) were invited via Prolific. To qualify for the study, they had to be currently working full-time as a manager and have regular interactions with subordinates at the time of the study. Those who had participated in any of our previous studies were also excluded. Owing to the COVID-19 pandemic (e.g. many people were laid off or furloughed), only 160 people met the criteria for inclusion in the study and completed the survey. Respondents were paid £2.50. We removed 17 participants from the analyses for failing to follow the communication task instructions. The final sample (*N* = 143) was 52% female with a mean age of 35.87 years (*SD =* 9.59), work experience of 16.67 years (*SD* = 9.56), tenure in their organization of 7.52 years (*SD* = 8.48), and 11.21 subordinates (*SD* = 32.42).

Participants were randomly assigned to a coping potential affirmation condition or a control condition. In both conditions, participants were asked to imagine themselves in the layoff scenario from Study 1a. However, we used a gender-neutral name (‘Taylor’) for the subordinate (see Hershcovis et al., 2017). Next, we introduced our manipulation. In the coping potential affirmation condition, participants read:

As Taylor’s manager, you will need to communicate the layoff decision to Taylor. Imagine that Taylor has agreed to meet you and you are now preparing for this meeting. While you are preparing, try to imagine that Taylor responds well to the way you are communicating the news during this meeting.

In the control condition, participants read: ‘As Taylor’s manager, you will need to communicate the layoff decision to Taylor. Imagine that Taylor has agreed to meet you and you are now preparing for this meeting.’ Following this manipulation, we measured anxiety. Finally, participants were instructed to communicate the layoff decision to Taylor.^6^ Following this task, participants completed filler items followed by our measure of core self-evaluations and a manipulation check.

### Measures

*Anxiety* was assessed with the same scale as in Study 1. *Interpersonal justice enactment* was rated by two independent coders following the same protocol as Study 1. The ICC_2_ (.83) indicated a high level of agreement. Discrepancies were identified and then resolved through discussion. The mean word count of the messages written by participants was 132.44 (*SD* = 68.67; min. = 23; max. = 345). *Core self-evaluations* were assessed with the same scale as Study 1. A *manipulation check* was used to assess the effectiveness of our affirmation manipulation on appraisals of coping potential (four items: ‘I was confident in my abilities to manage Taylor’s reaction’, ‘I questioned my ability to deliver the news effectively’ (reverse-coded), ‘I imagined Taylor reacting well to the way I was communicating the news’, ‘I imagined my own strengths about communicating the news to Taylor’; α = .60). The question stem was ‘**In preparing for the meeting with Taylor. . .’.** The response scale ranged from *not at all* (1) to *very much* (5).

### Results

Table 5 presents the means, standard deviations, reliabilities, and correlations. Our manipulation check indicated that participants in the coping potential affirmation condition (*M* = 3.34, *SD* = .76) appraised their coping potential more favorably than those in the control condition (*M* = 3.12, *SD* = .72); this effect was marginally significant, *t*(141) = −1.80, *p* = .074.

**Table 5.** Means, standard deviations, correlations, and reliabilities (Study 2).

Variable	*M*	*SD*	1	2	3	4
1. Core self-evaluations	3.48	.62	(.84)			
2. Manipulation^a^	.49	.50	.01	(–)		
3. Anxiety	4.52	1.35	−.30*	−.06	(.76)	
4. Interpersonal justice	2.90	.70	−.14^†^	−.05	.22*	(–)

*N =* 143 (coping potential affirmation condition: *n* = 70; control condition: *n* = 73). ^†^*p* < .10; *p < .01. Reliabilities are shown on the diagonal in parentheses.

a Conditions were coded as 1 (coping potential affirmation condition) versus 0 (control condition).

A CFA on our measures of core self-evaluations, anxiety, and interpersonal justice enactment indicated adequate fit, χ²(84) = 130.85, *p* < .001; CFI = .93; RMSEA = .06. Our proposed measurement model also had significantly better fit (*p* < .001) than an alternative model in which core self-evaluations and anxiety (i.e. the two self-reported variables) were combined, χ²(86) = 220.03, *p* < .001, providing evidence for the distinctiveness of our variables.

Before testing our hypotheses, we examined key bivariate relationships with linear regression. Consistent with our theorizing, core self-evaluations were negatively related to anxiety, *b* = −.65, *SE* = .18, *t*(141) = −3.70, *p* < .001, *R*^2^ = .09. Anxiety was positively associated with interpersonal justice enactment, *b* = .11, *SE* = .04, *t*(141) = 2.61, *p* = .010, *R*^2^ = .05.

Next, we turned to testing the interaction between core self-evaluations and our manipulation of coping potential (dummy-coded: coping potential affirmation condition = 1; control condition = 0) in predicting anxiety. The interaction was marginally significant; effect = .67, *SE* = .36, *t*(139) = 1.85, *p* = .066, Δ*R*^2^ = .02. In the control condition, as expected, core self-evaluations were negatively associated with anxiety; effect = −.90, *SE* = .22, *p* < .001. By contrast, this effect was not significant in the coping potential appraisals affirmation condition; effect = −.23, *SE* = .29, *p* = .42. This pattern of results is consistent with the manipulation for appraisals of coping potential ‘interrupting’ the relationship between core self-evaluations and anxiety. This provides evidence consistent with appraisals of coping potential mediating the relationship between core self-evaluations and anxiety. H2 was supported.

We then tested moderated mediation with core self-evaluations as the predictor, anxiety as the mediator, interpersonal justice enactment as the outcome variable, and our coping potential manipulation as a first-stage moderator (see Table 6 for the results). The index of moderated mediation (see Hayes, 2015) was marginally significant; index = .07, *SE* = .05, 90% CI [.00, .17]. As expected, the indirect effect of core self-evaluations on interpersonal justice enactment via anxiety was significant in the control condition (effect = −.09, *SE* = .05, 95% CI [−.19, −.01]) but not in the coping potential affirmation condition (effect = −.02, *SE* = .05, 95% CI [−.09, .04]), providing experimental evidence in support of a serial mediation. H3 was supported.

**Table 6.** Results of main regression analyses for Study 2.^a^

	Anxiety		Interpersonal justice enactment
	Model 1	Model 2	
Intercept	6.79** (.62)	7.73** (.78)	2.80** (.45)
Core self-evaluations	−.65** (.18)	−.90** (.22)	−.10 (.10)
Manipulation^b^		−2.50^†^ (1.27)	
Core self-evaluations x Manipulation^b^		.67^†^ (.36)	
Anxiety			.10* (.04)
*R* ^2^	.09	.11	.05

† *p* < .10; *p < .05; **p < .01. Values are unstandardized path coefficients with standard error estimates in parentheses.

a We did not conduct supplemental analyses for Study 2 because we did not have any control variables.

b Conditions were coded as 1 (coping potential affirmation condition) versus 0 (control condition).

By demonstrating the effect of ‘interrupting’ the mediation (i.e. by manipulating appraisals of coping potential), Study 2 provides evidence for the importance of appraisal processes (i.e. appraisals of coping potential and anxiety) in the relationship between core self-evaluations and the enactment of interpersonal justice. More precisely, our results indicated that in the control condition, core self-evaluations had a significant negative indirect effect on interpersonal justice enactment via anxiety. However, when appraisals of coping potential were affirmed, the indirect effect of core self-evaluations on interpersonal justice enactment via anxiety was non-significant. This suggests that core self-evaluations influence anxiety (and hence interpersonal justice enactment) *via* their effect on coping potential appraisals. Thus, by manipulating appraisals of coping potential, Study 2 provides experimental evidence in support of the hypothesized serial mediation, including the temporal ordering of the two mediators.

## Study 3

Study 3 tests our serial mediation model with appraisals of coping potential (measured rather than manipulated) and anxiety serving as sequential mediators in the relationship between core self-evaluations and the enactment of interpersonal justice (H3). We also use a modified methodology to further enhance the realism of the task used to assess interpersonal justice enactment. Whereas Studies 1 and 2 asked participants to communicate bad news to a fictitious subordinate, Study 3 asks participants to compose a message for one of their own subordinates. This allows our full serial mediation model to be tested in the context of an ongoing relationship.

### Participants and procedure

Full-time managers (*N =* 250) from the United Kingdom were recruited via Prolific and paid £2.50.^7^ Participants had to be currently working full-time as managers, have regular interactions with their subordinates (e.g. not furloughed), and not have previously participated in any of our studies. Twenty-four participants were removed from the analyses for failing to follow the communication task instructions. The final sample (*N* = 226) was 50% female with a mean age of 35.31 years (*SD =* 10.05), work experience of 16.50 years (*SD* = 10.00), tenure in their organization of 7.76 years (*SD* = 7.05), and 16.77 subordinates (*SD* = 38.30).

Participants were instructed to think of one employee in their organization who reports to them and to enter the first name (or a nickname) of this subordinate. To avoid biasing participants’ selection of a subordinate (e.g. to avoid participants selecting their least favorite subordinate for the purpose of this task), they were asked to report the name of a subordinate before receiving any specific information about the task. Next, participants received the following instructions:

At your organization, it has been decided that 10% of employees need to be laid off. [Name of subordinate] is among those being laid off. Imagine that you, as [name of subordinate]’s supervisor, need to communicate this news to [name of subordinate].

‘[Name of subordinate]’ was replaced with the actual name of the subordinate provided by the participant. Next, participants completed our measures of appraisals of coping potential and anxiety. They then received the following instructions: ‘You now need to communicate the layoff decision to [name of subordinate]. Please write down what you want to say. Imagine you are actually communicating the layoff decision to [name of subordinate].’ Following this task, participants completed filler items followed by our measure of core self-evaluations.

### Measures

*Appraisals of coping potential* were assessed with a scale from Chen et al. (2001) that was adapted to reflect appraisals related to the delivery of bad news to one’s subordinate. The prompt was: ‘Please think about your upcoming task of communicating the layoff decision to [name of subordinate]. Then indicate how much you agree with the following statements’ (six items: ‘I can cope with this task’, ‘I am confident that I can manage [name of subordinate]’s reaction’, ‘Compared to other people, I can do this task well’, ‘I am certain that I will accomplish this task effectively’, ‘I can succeed at conducting this task’, ‘I am confident that I can perform this task well’). The response scale ranged from *strongly disagree* (1) to *strongly agree* (5). *Anxiety* was measured with a four-item scale from Kouchaki and Desai (2015). The items were: anxious, worried, nervous, apprehensive. The prompt was ‘please respond to each statement below based on your feelings about communicating the news to [name of subordinate]’. The response scale ranged from *not at all* (1) to *very much* (7). *Interpersonal justice enactment* was rated by two independent coders following the same protocol as Study 1. The ICC_2_ (.80) indicated a high level of agreement. Discrepancies were identified and then resolved through discussion. The mean word count of the written messages was 83.13 (*SD* = 42.55; min. = 24; max. = 264). *Core self-evaluations* were assessed with the same scale as Studies 1 and 2.

*Subordinate gender* was assessed to examine whether the recipient’s gender impacted the enactment of interpersonal justice (see Varty et al., 2020). Participants were asked to report their *subordinate’s gender* (‘What is [name of subordinate]’s gender?’; *male* = 0, *female* = 1).

Given that supervisors were asked to communicate bad news to one of their own subordinates, we assessed *leader–member exchange* using a contextualized version of Liden and Maslyn’s scale (1998; 11 items; e.g. ‘I like [name of subordinate] very much as a person’; ‘I respect [name of subordinate]’s knowledge and competence on the job’). Notably, this scale assesses the extent to which the supervisor–subordinate relationship is based on mutual trust, respect, and liking, including respect for the subordinate’s professional skills and knowledge. The professional respect items relate to the subordinate’s performance, which may be especially important in a layoff context (i.e. managers may feel differently about letting go low versus high performers). Participants indicated how much they agreed with each statement ‘in general’. Response scales ranged from *strongly disagree* (1) to *strongly agree* (5).

### Results and discussion

Table 7 presents the means, standard deviations, reliabilities, and correlations. A CFA on our measures of core self-evaluations, appraisals of coping potential, anxiety, and interpersonal justice enactment indicated good fit, χ²(180) = 308.03, *p* < .001; CFI = .94; RMSEA = .06. We then compared our model to alternative measurement models, including a model in which core self-evaluations, appraisals of coping potential, and anxiety (i.e. the three self-report variables) were combined (χ²(185) = 690.52, *p* < .001), a model in which appraisals of coping potential and anxiety were combined (χ²(183) = 430.24, *p* < .001), a model in which core self-evaluations and appraisals of coping potential were combined (χ²(183) = 582.77, *p* < .001), and a model in which core self-evaluations and anxiety were combined (χ²(183) = 485.62, *p* < .001). In every case, our proposed measurement model had significantly better model fit than alternative models (*p*s < .001), providing evidence for the distinctiveness of our variables.

**Table 7.** Means, standard deviations, correlations, and reliabilities (Study 3).

Variable	*M*	*SD*	1	2	3	4	5	6
1. Core self-evaluations	3.40	.70	(.87)					
2. Appraisals of coping potential	3.80	.83	.34**	(.90)				
3. Anxiety	4.82	1.46	−.37**	−.55**	(.92)			
4. Interpersonal justice	3.00	.85	−.07	−.08	.19**	(–)		
5. Subordinate gender	.50	.50	−.01	−.06	.13*	.04	(–)	
6. Leader–member exchange	3.75	.86	−.07	−.05	.16*	.15*	.08	(.94)

*N* = 226. *p < .05; **p < .01. Reliabilities are shown on the diagonal in parentheses. Gender is dummy coded male = 0, female = 1.

Table 8 displays the results of the main and supplemental analyses. Core self-evaluations were positively associated with appraisals of coping potential, *b* = .40, *SE* = .08, *t*(224) = 5.34, *p* < .001, *R*^2^ = .11. Core self-evaluations were negatively related to anxiety, *b* = −.78, *SE* = .13, *t*(224) = −5.97, *p* < .001, *R*^2^ = .14. Appraisals of coping potential were also negatively associated with anxiety, *b* = −.97, *SE* = .10, *t*(225) = −9.85, *p* < .001, *R*^2^ = .30. Anxiety was positively related to interpersonal justice enactment, *b* = .11, *SE* = .04, *t*(225) = 2.26, *p* = .004, *R*^2^ = .04.

**Table 8.** Results of main and supplemental regression analyses for Study 3.

	Appraisals of coping potential		Anxiety		Interpersonal justice enactment	
	Model 1	Model 2	Model 1	Model 2	Model 1	Model 2
*Main analysis*						
Intercept	2.44** (.26)		9.53** (.47)		2.30** (.56)	
Core self-evaluations	.40** (.08)		−.44** (.12)		−.02 (.09)	
Appraisals of coping potential			−.85** (.10)		.04 (.08)	
Anxiety					.12* (.05)	
*R* ^2^	.11		.34		.04	
*Supplemental analysis*						
Intercept	4.04** (.26)	2.58** (.37)	3.47** (.44)	8.31** (.61)	2.39** (.26)	1.87** (.59)
Subordinate gender	−.09 (.11)	−.09 (.11)	.35 (.19)	.27 (.16)	.05 (.11)	.02 (.11)
Leader–member exchange	−.05 (.07)	−.02 (.06)	.31** (.11)	.24* (.09)	.16* (.07)	.13 (.07)
Core self-evaluations		.40** (.08)		−.39** (.12)		−.01 (.09)
Appraisals of coping potential				−.84** (.10)		.05 (.08)
Anxiety						.10* (.05)
*R* ^2^	.01	.12	.05	.37	.03	.05

*p < .05; **p < .01. Values are unstandardized path coefficients with standard error estimates in parentheses. The supplemental analysis was performed using control variables. Gender is dummy coded male = 0, female = 1.

While our objective in Study 3 was to focus on the serial mediation, we also re-tested H1 and H2. H1 proposes that core self-evaluations have a negative indirect effect on interpersonal justice enactment via anxiety. The indirect effect was significant; indirect effect = −.08, SE = .04, 95% CI [−.16, −.02]. H1 was supported. For H2, the indirect effect of core self-evaluations on anxiety via appraisals of coping potential was significant; indirect effect = −.34, *SE* = .07, 95% CI [−.49, −.20]. The mediation model accounted for 34.14% of the variance in anxiety. H2 was supported. Results for the serial mediation model indicated that the indirect effect of core self-evaluations on interpersonal justice enactment via appraisals of coping potential and anxiety was significant; indirect effect = −.04, *SE* = .02, 95% CI [−.08, −.01]. The mediation model accounted for 3.71% of the variance in the enactment of interpersonal justice. H3 was supported.

We also examined the influence of the subordinate’s gender and leader–member exchange. However, all hypothesized effects remained substantively similar and significant when these variables were included in the model as covariates (see Table 8).

Taken together, Study 3 provides further empirical support for our argument that core self-evaluations are negatively related to the enactment of interpersonal justice via appraisals of coping potential and anxiety. Moreover, these effects replicated with a protocol involving the supervisor’s own subordinate and with leader–member exchange ruled out as an alternative explanation, thereby providing further confidence in our findings.

## General discussion

Our findings indicate that core self-evaluations can detract from the enactment of interpersonal justice. Further, our studies showed that these effects occur via appraisal processes and that anxiety is important for motivating the enactment of interpersonal justice. Below, we discuss the implications of these findings.

### Core self-evaluations and the enactment of justice

We identified core self-evaluations as an important antecedent that can negatively influence the enactment of interpersonal justice. While core self-evaluations are often touted as being a significant contributor to managerial effectiveness (e.g. Erez and Judge, 2001), our studies indicate that core self-evaluations may undermine a critical managerial behavior – the enactment of interpersonal justice. This insight adds to the emerging literature indicating that higher core self-evaluations do not always translate into more positive outcomes (e.g. Grant and Wrzesniewski, 2010; Hiller and Hambrick, 2005; Stajkovic, 2006). This suggests that high core self-evaluations are not a panacea for ensuring managerial effectiveness. Instead, managers with high core self-evaluations may be especially susceptible to failing to uphold interpersonal justice.

Further, our studies contribute to the core self-evaluations literature by highlighting a novel outcome for core self-evaluations (i.e. interpersonal justice enactment) and empirically testing the processes through which this personality trait can influence behavior (i.e. appraisals of coping potential and anxiety). Thus, our research responds to calls to examine outcomes of core self-evaluations beyond job satisfaction and performance as well as the mechanisms through which these outcomes occur (e.g. Chang et al., 2012). Importantly, our findings indicate that core self-evaluations can have unintended effects by influencing appraisal processes and undermining behaviors that are important for social relationships. Future research should further examine the contexts in which core self-evaluations may (unintentionally) undermine versus facilitate one’s behaviors and social relationships as well as identify boundary conditions for these effects.

### The importance of appraisal processes

Our studies also empirically support the theoretical notion that core self-evaluations are negatively related to the enactment of interpersonal justice because of their impact on appraisal processes (i.e. appraisals of coping potential and the elicitation of anxiety). These findings are theoretically important for several reasons. First, although the justice literature has recognized that some people are less likely to enact justice behaviors, *why* these effects occur has remained elusive. Drawing on appraisal theories, our studies identify a novel managerial characteristic that can impact the enactment of interpersonal justice (i.e. core self-evaluations) and highlight how this relationship can be understood through the lens of appraisal theory. Future research may benefit from identifying additional managerial characteristics that can facilitate or hinder the enactment of justice, boundary conditions associated with these effects, and other processes through which managerial characteristics may impact the enactment of justice.

Second, by enhancing our theoretical understanding of *why* actors may be motivated to enact justice, our studies also answer calls to understand how to motivate justice behaviors (e.g. Ambrose and Schminke, 2009). Although descriptive accounts of how justice behaviors can be motivated by cognition and emotions have been offered (e.g. Scott et al., 2009, 2014), appraisal theories provide nuanced insights into how cognitions can precede and influence the emergence of emotions. These insights are critical for understanding and motivating justice behaviors.

Third, although the justice literature has focused on training managers on the importance of enacting justice (Skarlicki and Latham, 1996), our findings highlight that it is also critical to recognize that characteristics of the actor can influence their motivations and willingness to deliver justice (i.e. justice can be a motivated phenomenon; Barclay et al., 2017). Interventions seeking to enhance the enactment of justice may therefore benefit from educating managers on when and why they may be less likely to enact justice as well as how to overcome these effects.

### Beyond valence: The importance of discrete emotions

Our research responds to numerous calls in the justice literature to examine the role and impact of discrete emotions, especially emotions other than anger (e.g. Barclay and Kiefer, 2019; Cropanzano et al., 2011, 2017). While anger is often emphasized because of its association with negative outcomes for the organization (e.g. retaliation), scholars have argued that it is also imperative to examine emotions that are integral to individuals’ lived experiences (e.g. Cropanzano et al., 2017). Importantly, our studies indicate that anxiety is an important motivating force for the enactment of interpersonal justice. Moreover, anxiety *increases* the enactment of interpersonal justice, which is a stark contrast to the *decrease* previously observed for anger (see Scott et al., 2014). These findings highlight the importance of emotions for motivating justice behaviors and challenge the assumption that negative emotions are always dysfunctional for the enactment of justice. Further, the contrasting effects for anger and anxiety in propelling the enactment of interpersonal justice highlight the importance of recognizing conceptual distinctions between discrete emotions.

Traditionally, the justice literature has positioned negative emotions as dysfunctional and recommended curtailing these emotions (e.g. Scott et al., 2014). By contrast, appraisal theories highlight that negative emotions are adaptive for individuals who experience the emotion but may or may not be functional for the organization or others. For example, anxiety may be functional for the organization and bad news recipients by promoting interpersonal justice whereas anger may be dysfunctional by prompting aggressive behaviors (e.g. Barclay et al., 2005). Thus, it is critical to recognize why discrete emotions arise (i.e. their adaptive function for the person who is experiencing the emotion) as well as the degree to which the emotion is (dys)functional for the organization and other stakeholders.

### Strengths, limitations, and future research opportunities

Our hypotheses were generally supported across three studies using four samples (three samples with only managers and one sample with managers and full-time employees), two different bad news contexts (i.e. a layoff and a promotion denial), disparate methodologies (e.g. manipulated versus measured appraisals of coping potential, fictional versus actual subordinate), and with studies collected prior to and during the pandemic. Importantly, our package of studies also included a moderation of process design (Study 2) that provides evidence for the causal ordering of proposed model. Nonetheless, future research may wish to further examine potential endogeneity issues (see Antonakis et al., 2010).

Although Study 2 may have been underpowered owing to the difficulties associated with recruiting eligible participants during the early stages of the COVID-19 pandemic, the consistent replication of findings across studies provides confidence in these findings. Our studies also ruled out alternative explanations (e.g. sense of power, dark triad, trait empathy, leader-member exchange, prior experiences with layoffs, supervisory position, and subordinate gender), which provides further confidence in the robustness of our findings. Nonetheless, future research may benefit from further exploring moderators of these effects, such as relationship continuity (e.g. anxiety may be heightened and/or have stronger effects when the relationship is likely to continue). Future research may also benefit from further exploring interventions that target appraisals of coping potential. More precisely, our manipulation in Study 2 showed that visualizing that the meeting with the subordinate would go well resulted in less anxiety and therefore less enactment of interpersonal justice. While this manipulation reflects visualization techniques that are often advocated to enhance performance (e.g. Driskell et al., 1994), our study highlights when this technique may backfire. However, it is important to note that this manipulation may have not only impacted appraisals of coping potential but also perceived threat. Indeed, the reliability for our manipulation check was low, suggesting that future research may benefit from re-examining these relationships.

We tested our hypotheses in a controlled environment to enhance internal validity and rule out alternative explanations (e.g. Aronson and Carlsmith, 1968). This also allowed behavioral responses (i.e. communications) to be assessed while ensuring that no recipients of bad news were harmed by failing to receive justice. By examining our hypotheses with samples of full-time managers and employees, we ensured that these issues had relevance to the sample. Further, we tried to enhance psychological realism by using stimuli that provided contextual information (Studies 1 and 2) or by using the names of actual subordinates (Study 3). Although these strategies may have introduced noise into the studies, the consistent replication of the results across multiple studies can enhance confidence in the findings.

While our findings highlighted the facilitative effect of anxiety, previous research has shown that anxiety can have facilitative or debilitating effects depending on the situation (e.g. Carver and Scheier, 1988; Cheng and McCarthy, 2018). We argued that anxiety can have facilitative effects while delivering bad news because the individual can experience enhanced arousal and motivation in this social situation. Further, there is a clear behavior that is likely to be effective in reducing potential harm (i.e. the enactment of interpersonal justice). However, anxiety may be debilitating under different circumstances (e.g. when it is unclear how to address the potential for harm) or at different stages of the process (e.g. anxiety may prompt people to avoid the recipient prior to delivering the bad news). Future research should further explore the role of moderators that can influence when anxiety is facilitative versus debilitating.

We examined anxiety related to the delivery of bad news. Interestingly, while 80% of the population reported enhanced generalized anxiety during the COVID-19 pandemic (Roy et al., 2020), the amount of anxiety about delivering bad news was similar in the samples collected prior to (*M* = 4.63 and 4.95) and during the pandemic (*M* = 4.52 and 4.82; see Tables 1, 3, 5, and 7). This highlights the importance of distinguishing between generalized versus targeted anxiety. Moreover, it is important to note that anxiety that is targeted at the situation may have disparate effects compared to generalized moods (e.g. generalized anxiety that lacks a target) or incidental emotions (e.g. anxiety that is not targeted at the situation) (see Hillebrandt and Barclay, 2017). For example, generally feeling anxious may be associated with avoidance rather than approach tendencies because it is less clear what is needed to relieve this feeling. Future research should further examine the disparate downstream implications related to emotion targets.

We focused on the enactment of interpersonal justice because it is considered the most critical form of justice during the *delivery* of bad news (Bies, 2013). By contrast, other forms of justice may be more important during other phases. For example, procedural justice may be especially important as actors engage in preparation activities (e.g. identifying the criteria to decide who will be laid off). Future research may benefit from identifying actor characteristics that may influence the enactment of other forms of justice and/or other justice rules that may be critically important for perceptions of fair treatment (e.g. Fortin et al., 2020). Further, we focused on *how* people delivered the news (i.e. with interpersonal sensitivity by enacting interpersonal justice). Future research may also wish to examine *what* is being communicated (e.g. information related to distributive, procedural, or informational justice). Future research may also benefit from examining moderators of people’s desire to be perceived as fair (e.g. moral identity).

People may appraise situations in ways that elicit other emotions. For example, shame or embarrassment may be elicited if people believe that they are personally responsible for the negative outcome (e.g. Lazarus, 1991). This may prompt distancing instead of approach behaviors (e.g. Folger and Skarlicki, 2001). Future research should also further examine how individuals may differentially appraise situations (Lazarus, 1991). Different discrete emotions may also be differentially relevant for the various forms of justice. For example, social and moral emotions may be more relevant for facilitating interpersonal justice because this form of justice is about interpersonal (i.e. social) relationships and has a moral basis (Bies, 2001, 2013). By contrast, anger may be influential for informational justice because it can encourage actors to provide more thorough explanations (Scott et al., 2009). Guilt may be important for procedural justice, especially during the preparation stage, because it can propel actors to approach their environment (e.g. Lazarus, 1991). That is, guilt may encourage actors to spend the time, effort, and persistence needed to uphold procedural justice (e.g. by ensuring that accurate information is identified, gathered, and used). Thus, it is critical to further explore the effects of discrete emotions and how they may differentially influence the enactment of other forms of justice.

### Practical implications

Our findings offer important practical insights. Previous research has shown that core self-evaluations are positively associated with a broad array of behaviors that typically enhance managerial effectiveness (e.g. goal setting, persistence, leadership behaviors; Erez and Judge, 2001; Resick et al., 2009). However, core self-evaluations can *negatively* influence interpersonal justice enactment. This suggests that it is important not to assume that core self-evaluations universally predict positive behaviors. Instead, this trait may create challenges for certain behaviors (e.g. the enactment of interpersonal justice). This possibility is important to recognize given that enacting interpersonal justice is associated with critical outcomes related to employee, manager, and organizational effectiveness (for a meta-analytic review, see Colquitt et al., 2013).

Importantly, our findings indicated that managers’ anxiety can prompt the enactment of interpersonal justice. While scholars have suggested that organizations should reduce managers’ negative affect to increase the enactment of interpersonal justice (e.g. Scott et al., 2014), our findings indicate that anxiety can encourage managers to enact interpersonal justice. This suggests that it is important not to assume that all negative emotions operate in a similar manner. Instead, it is critical to consider the functions of disparate negative emotions to understand their effects. In the case of anxiety, our findings indicate that this ‘negative’ emotion may have motivational and facilitative effects for the enactment of interpersonal justice. That is, anxiety may signal to managers that this is a situation where enacting interpersonal justice is important. Thus, we encourage managers to recognize how disparate negative emotions may influence their enactment of interpersonal justice, including how and when these emotions may have facilitating versus hindering effects. Doing so may also enable managers to leverage negative emotions that can promote justice while suppressing negative emotions that can hinder these behaviors.

Given that core self-evaluations were negatively associated with the enactment of interpersonal justice, this raises the question of what managers and/or organizations can do to overcome these effects. First, training programs to enhance justice are likely to benefit from going beyond a focus on the importance of enacting justice to also include who is less likely to enact justice and why these effects may occur. Second, previous research has identified strategies that can be effective for overcoming dispositional tendencies. For example, the enactment of interpersonal justice when delivering bad news may be enhanced by increasing actors’ self-awareness by placing them in self-focusing situations (e.g. in front of a mirror; Whiteside and Barclay, 2016) or by increasing their awareness that they may be less inclined to enact justice (e.g. Whiteside and Barclay, 2018). Thus, organizations and/or managers should recognize *who* is less likely to enact justice and consider implementing strategies to overcome these effects.

## Conclusion

Our studies point to the importance of recognizing that managerial characteristics can impact the enactment of interpersonal justice and that anxiety can facilitate the enactment of interpersonal justice. We encourage scholars to further examine the factors that can motivate the enactment of justice since the strides that are made in answering the question of ‘what motivates people to deliver justice?’ are critical for enhancing our theoretical understanding and practical ability to promote justice in the workplace.

## Appendix

### Study 1b scenario

*Please read the scenario below. Imagine that the events are really happening to you. Please make sure you take your time and read the scenario carefully.*

Imagine you are a manager in an organization. You need to tell one of your employees, Tom, that he will not receive the promotion he expected.

The entry-level positions in your department are not particularly well paid and the work in these positions can be tedious for employees, particularly those with high potential. However, high-performing employees in your department have typically been promoted quickly into more senior positions that come with more interesting and varied tasks, along with more flexible work hours and a 20% salary increase.

Promotion decisions in your organization are made by an independent ‘Evaluation Committee’ and are based on (a) employee seniority and (b) employee performance evaluations.

Earlier this year, you encouraged two of your subordinates, Mark and Tom, to apply for the promotion. Having worked with Mark and Tom for several years, you were confident that they would both get promoted. In fact, you had told them they could count on getting promoted if they maintained their productivity levels, which they did.

Mark has worked in your department for 41 months. On his first annual performance review, Mark was commended for his good performance, which he has maintained ever since. Tom has worked in your department for 36 months. On his first annual performance review, Tom was criticized for frequently being late for meetings. However, his second performance review indicated improved punctuality and professionalism. In Tom’s last review, he was commended for his excellent effort and performance.

Unfortunately, sales in your company have declined this year, following the loss of several large accounts to competitors. To offset declining revenues, upper management has decided to freeze salaries and restrict promotions. The Chair of the Evaluation Committee has informed you that due to these conditions, only one employee could be promoted and that Mark has been selected as the successful candidate.

You are aware that Tom’s initial work performance may have suffered because he was still in school part-time. You also know that Tom recently put a down-payment on a house. However, you agree that freezing salaries and limiting promotions is vital to staying in business and getting back on track. Unfortunately, because of Tom’s lower level of seniority and mixed performance reviews, he will not be promoted and will not receive a salary increase. There is less than a 50% chance that Tom will be promoted next year or that other attractive opportunities within the company will become available for him in the foreseeable future.

*Please imagine that you, as Tom’s manager, need to communicate the news to Tom.*

## Notes

1 Anxiety can be distinguished from fear, which reflects ‘immediate, concrete, and overwhelming physical danger’ (Lazarus, 1991: 122; also see Marks, 1987; Perkins et al., 2007). Further, whereas fear motivates a rapid flight response, anxiety enhances vigilance (e.g. Mathews, 1990; Rachman, 2004).

2 We did not conduct a priori power sample size analyses for Study 2 (moderation of process design) or Study 3 (serial mediation involving measured variables) given the difficulties associated with estimating the volume of parameters needed for an a priori analysis and the issues associated with quantifying the magnitude of the effects and their product (for detailed discussions, see Hayes, 2018; Schoemann et al., 2017).

3 Five participants failed one attention check. However, none failed both checks and our results were substantively similar when participants who failed an attention check were excluded from the analyses. In all subsequent studies, excluding versus including participants who failed at least one attention check resulted in substantively similar results. Thus, we did not exclude participants owing to potential inattentiveness from any analyses.

4 For all studies, we modeled core self-evaluations (CSE) following Judge et al.’s (2003) approach. Specifically, each item of the CSE scale was loaded on one of four factors (i.e. self-esteem, self-efficacy, locus of control, neuroticism) that the item best indicated. Then, given that the CSE scale is designed to tap into an underlying CSE construct rather than four distinct traits, the six covariances among the four factors were fixed to 1. Interpersonal justice enactment was included in the model as a single-item (observed) variable.

5 We conducted our analyses with Process instead of structural equation modeling (SEM). We chose Process because simulation studies have shown that Process either results in *less* biased or similar findings to SEM when observed variables and moderate sample sizes are used – these conditions are reflected in our studies (see Hayes, 2018; Hayes et al., 2017).

6 According to Lazarus (1991: 238), appraisals of coping potential reflect people’s assessments of their perceived ability to manage or ameliorate a threatening situation and can be enhanced by ‘cognitive coping efforts to think positively’. Thus, our manipulation focused on encouraging people to think positively about the situation (versus a control condition). While this manipulation was intended to target appraisals of coping of potential, it is possible that this may have also decreased the perceived threat associated with the situation.

7 This study was collected during the COVID-19 pandemic. At the time of data collection, Prolific had recently updated their pre-screening protocols. This enabled managers who were currently working full-time with regular subordinate interactions (e.g. not laid off or furloughed) to be directly recruited.
